# Electrospray Beta-Glucan Particle Coated PVP/CA Electrospun Mat as a Potential Scaffold for Dental Tissue Regeneration

**DOI:** 10.3390/polym17192693

**Published:** 2025-10-05

**Authors:** Thanutham Somboonchokephisal, Pratchaya Tipduangta, Sarawut Kumphune, Tanida Srisuwan

**Affiliations:** 1 Department of Restorative Dentistry and Periodontology, Faculty of Dentistry, Chiang Mai University, Chiang Mai 50200, Thailand; thanutham.som@mfu.ac.th; 2 School of Dentistry, Mae Fah Luang University, Chiang Rai 57100, Thailand; 3 Department of Pharmaceutical Sciences, Faculty of Pharmacy, Chiang Mai University, Chiang Mai 50200, Thailand; pratchaya.t@cmu.ac.th; 4 Biomedical Engineering and Innovation Research Center, Chiang Mai University, Mueang Chiang Mai District, Chiang Mai 50200, Thailand; sarawut.kumphune@cmu.ac.th; 5 Biomedical Engineering Institute (BMEI), Chiang Mai University, Mueang Chiang Mai District, Chiang Mai 50200, Thailand

**Keywords:** polysaccharides, preparation method, carbohydrates, paramylon, biocompatibility, drug delivery system, regenerative medicine, dentistry, regenerative endodontic, stem cells

## Abstract

Regenerative endodontic procedures (REPs) are a promising treatment for immature teeth with pulpal necrosis. However, the outcomes remain unpredictable, partly due to scaffold limitations. Beta-glucan (BG), a bioactive polysaccharide with regenerative properties, may enhance scaffold performance. This study aimed to fabricate BG-coated polyvinylpyrrolidone/cellulose acetate (PVP/CA) electrospun scaffolds and evaluate their physicochemical properties and cell attachment. Electrospun scaffolds were fabricated by electrospinning a 10% *w*/*v* PVP/CA (70:30) solution in acetone and N,N-dimethylacetamide (2:1) (PC). BG (8% *w*/*v* in 1 M NaOH) was electrosprayed onto the scaffold at 0.1, 0.2, and 0.4 mL volumes, generating PC-BG01, PC-BG02, and PC-BG04, respectively. Scaffold characterization included SEM, FTIR, BG enzymatic assay, water absorbance, degradation, and cell adhesion assays. SEM images of the scaffolds exhibited smooth cylindrical fibers (547.3–585.9 nm diameter) with high porosity (42.37–49.91%). BG particles were confirmed by elemental analysis and BG enzymatic assay. At 28 days, the PC group showed significant fiber diameter and porosity reduction. BG particle degradation was observed at 14 and 28 days. Notably, BG-coated scaffolds significantly enhanced initial apical papilla cell adhesion at 1 and 24 h. These findings highlight the potential of BG-coated scaffolds as bioactive scaffolds for REPs.

## 1. Introduction

Regenerative endodontic procedures (REPs) are biologically based treatment strategies primarily developed for managing immature permanent teeth with necrotic pulp tissue. The goal is to restore dental pulp tissue containing vascular and neural components essential for continued root development and tooth function [1]. This regenerative approach follows the principles of tissue engineering, relying on three key elements: stem cells, signaling molecules, and scaffolds. Clinically, REPs have demonstrated favorable outcomes in many cases [2,3,4,5,6]. However, treatment results remain unpredictable in certain instances, with some cases showing no evidence of continued root development or presenting excessive calcification [5,7]. Additionally, histological analyses of treated teeth have revealed undesirable outcomes, including the formation of tissue that does not resemble native pulp. These limitations highlight the need for continued advancements in biologically driven approaches to improve treatment predictability and enhance regenerative outcomes.

Scaffolds are one of the key components involved in tissue regeneration [8]. In the context of REPs, various scaffold types have been employed, with the most commonly used being the natural scaffold formed from the patient’s own blood during treatment [9,10]. However, this approach presents several limitations, including the unpredictable formation of a blood clot, unquantified levels of active signaling molecules, and variable clot quality. Although alternative scaffold sources have been introduced, an optimal scaffold material has yet to be identified. A recent meta-analysis investigating scaffold types used in REPs reported no significant differences in clinical outcomes among the currently available materials, most of which are natural scaffolds [11,12]. These findings underscore the need for further development of synthetic scaffolds that incorporate bioactive components, offer ease of use, and possess the capacity to promote targeted tissue regeneration.

BG, a naturally derived polysaccharide, has attracted significant attention for its diverse bioactivities relevant to wound healing and tissue regeneration. Multiple studies have demonstrated that beta-glucan enhances immune cell activation, reparative cell proliferation and migration, angiogenesis, and collagen synthesis, supporting its therapeutic potential in medically regenerative applications [13,14,15,16,17,18]. Notably, beta-glucan has also been shown to decrease the collagen type I/type III ratio in dermal wound, a shift that may reduce the risk of intracanal calcification—a common complication following regenerative endodontic procedures [19]. Recent research additionally reports beta-glucan’s ability to promote odontogenic differentiation while modulating inflammatory responses, highlighting its potential in dental tissue engineering [20]. These findings form the basis for our interest in incorporating beta-glucan into a user-friendly scaffold system suitable for delivery within the narrow confines of the root canal space.

PVP and CA are biocompatible synthetic polymers that have been widely studied as composite materials for electrospun scaffolds, particularly for the delivery of water-insoluble drugs due to their ability to improve solid dispersion [21,22]. PVP is a highly hydrophilic polymer that dissolves rapidly in aqueous environments, allowing for controlled drug release and surface activity [23]. In contrast, CA, a semi-synthetic polymer derived from the acetylation of cellulose—a polysaccharide found in plant cell walls—exhibits low water solubility and slow biodegradability, making it suitable as a structural support within the fiber matrix [24]. PVP/CA blended composite scaffold typically exhibit a core–shell structure, with CA forming the core and PVP migrating to the outer shell. This distribution is driven by the lower surface energy of PVP, which causes it to orient toward the fiber surface during electrospinning [25,26]. Previous studies have explored PVP/CA nanofibers for tissue regeneration applications [25,27]. One report observed hydroxyapatite formation on PVP/CA mats, supporting their potential application in bone regeneration [25]. Since hydroxyapatite formation is also a key initial event in dental hard tissue regeneration, this finding may have relevance for REPs scaffold development.

This study aimed to develop, optimize, and characterize a BG-coated scaffold based on PVP/CA blended composite electrospun scaffold for application in REPs, with the design guided by key criteria for an ideal scaffold [28,29,30].

## 2. Materials and Methods

### 2.1. Materials

Beta-glucan (β-1,3-glucan from Euglena gracilis, average M_n_ = 500,000 g/moL), cellulose acetate (CA, average M_n_ = 30,000 g/mol), dispase II, α-minimum essential medium (α-MEM), fetal bovine serum (FBS), penicillin-streptomycin, 4′,6-diamidino-2-phenylindole dihydrochloride (DAPI), paraformaldehyde, and L-ascorbic acid were obtained from Sigma-Aldrich (St. Louis, MO, USA). Polyvinylpyrrolidone (PVP, average M_n_ = 360,000 g/mol) was purchased from Tokyo Chemical Industry Co., Ltd. (Tokyo, Japan). Acetone (analytical grade) was obtained from RCI Labscan Ltd. (Bangkok, Thailand), and N,N-dimethylacetamide (DMAc, analytical grade) was obtained from Fisher Scientific (Loughborough, UK). Collagenase I was purchased from Gibco (Gaithersburg, MD, USA). AlamarBlue cell viability reagent was obtained from Invitrogen (Carlsbad, CA, USA). The enzymatic yeast β-glucan assay kit was purchased from Neogen (Lansing, MI, USA). Triton X-100 was obtained from USB Corporation (Cleveland, OH, USA).

### 2.2. Scaffold Fabrication

#### 2.2.1. Electrospinning of Blended PVP/CA Scaffold

PVP and CA were used to prepare a blended composite polymer solution. The PVP and CA powders were mixed in ratio of 30:70 by weight and dissolved in a solvent mixture of acetone and N,N-dimethylacetamide (DMAc) at a ratio of 2:1 (*v*/*v*) with final polymer concentration of 10% (*w*/*v*).

The blended PVP/CA composite scaffold was fabricated using the electrospinning technique. The polymer solution was loaded into a 10 mL plastic syringe equipped with a blunt 18G stainless steel needle (inner diameter: 0.8 mm). Electrospinning was performed onto a rotating drum collector (12 cm in diameter, 30 cm in length), covered with household-grade aluminum foil and positioned 15 cm from the needle tip. The drum rotation speed was set at 250 rpm. An ES40P-20 W high-voltage power supply (Gamma High Voltage Research, Ormond Beach, FL, USA) and an automated syringe pump New Era NE-300 (Pump Systems Inc., Franklin, NH, USA) were used to maintain a flow rate of 1.5 mL/h and an applied voltage of 15 kV. The process was carried out under ambient conditions of 25 ± 2 °C and 50 ± 5% relative humidity.

#### 2.2.2. Incorporation of Beta-Glucan into the Scaffold

Beta-glucan powder was dissolved separately in 1 M sodium hydroxide (NaOH) in deionized water to obtain a stock solution with a final concentration of 8% (*w*/*v*). The prepared beta-glucan stock solution was electrosprayed onto the PVP/CA scaffold. The solution was dispensed using a separate 10 mL plastic syringe fitted with a blunt 18G stainless steel needle. Electrospraying was performed onto an aluminum foil-covered collector placed 10 cm from the needle tip, with a rotation speed of 250 rpm. The flow rate control by an NE-300 syringe pump and applied voltage by an ES40P-20 W high-voltage power supply were maintained at 0.8 mL/h and 20 kV, respectively, under the same ambient environmental conditions (25 ± 2 °C, 50 ± 5% RH). Based on our preliminary biocompatibility results (Figure S1), which indicated cytotoxicity at higher BG deposition (>0.5 mL), BG solution was electrosprayed at different volumes: 0.1, 0.2, and 0.4 mL, designated as PC-BG01, PC-BG02, and PC-BG04, respectively. These scaffold formulas were then evaluated for characterization in comparison with the uncoated PVP/CA (PC) scaffold. The specific volumes used for each formulation are summarized in Table 1.

### 2.3. Scaffold Morphology

The scaffold morphology was observed using scanning electron microscopy (SEM, TESCAN Vega IV, Brno, Czech Republic). Samples were coated with gold using a sputter coater, and imaging was performed at an accelerating voltage of 15 keV. Quantitative measurements, including fiber diameter and porosity (defined as the percentage of area not covered by fibers), were analyzed using SEM images taken at 30.0k× magnification with ImageJ software [31]. To confirm the presence of beta-glucan on the scaffold surface, regions suspected of beta-glucan deposition were further analyzed using energy-dispersive X-ray spectroscopy (EDS, Xplore 30 Oxford Instruments, Oxfordshire, UK).

### 2.4. Fourier-Transform Infrared (FTIR) Spectroscopy

The scaffolds were prepared into 1 × 1 cm square for acquiring FTIR spectra from a BrukerIF-66 spectrometer (Bruker Optics, Coventry, UK) equipped with a Golden gate MKII accessory from Specac Ltd. (Orpington, UK). Spectra were collected with a data resolution of 2 cm^−1^, taking 32 scans in the range of 4000–400 cm^−1^ to evaluate the chemical characteristics of the samples. The raw materials, which are polymer powder (BG, PVP, and CA), were also evaluated as a control to identify characteristic functional group peaks of the original. OPUS version 8.7.41 (BrukerOptics, Coventry, UK), a spectral analysis software, was used to interpret the data.

### 2.5. Beta-Glucan Quantitative Analysis

The amount of beta-glucan incorporated into the scaffolds was quantified using the Enzymatic Yeast β-Glucan Assay Kit, following the manufacturer’s protocol. Briefly, 20 mg of each scaffold sample was finely cut using sterile scissors and then hydrated in 2 M potassium hydroxide (KOH) with continuous stirring for 30 min in an ice bath to solubilize beta-glucan from the scaffold. Following solubilization, 40 μL of β-glucan–specific enzyme mixture (Glutamix) and 1.6 mL of 1.2 M sodium acetate buffer (pH 3.8) were added to each sample, which was then incubated at 40 °C for 16 h to hydrolyze β-1,3-glycosidic bonds of the backbone of beta-glucan, yielding a glucose monomer as the end product [32]. After incubation, 10 mL of distilled water was added to the sample, mixed, and then centrifuged at 3000 rpm for 10 min. The supernatant was collected, and glucose content was determined by adding glucose oxidase/peroxidase reagent. Absorbance was measured at 510 nm using a microplate reader (Tecan Trading AG, Männedorf, Switzerland). The assay included a reagent blank (0.1 mL of 200 mM sodium acetate buffer, pH 5.0 + 4.0 mL of glucose oxidase/peroxidase reagent) and a D-glucose standard (0.1 mL of D-glucose solution, 1.5 mg/mL + 4.0 mL of the reagent). Beta-glucan content (% *w*/*w*) was calculated using the following formula:

Beta-glucan (%*w*/*w*) = ΔA × (F/W) × 10.836 (1)

In this equation, ΔA represents the absorbance difference between the reaction and the sample blank, F is the conversion factor from absorbance to micrograms of glucose (calculated from D-glucose standard measurement), and W is the weight of the analyzed scaffold sample in milligrams. This calculation provides the percentage of beta-glucan relative to the scaffold weight.

### 2.6. Water Absorption Capacity

Water absorption capacity was evaluated by calculating the percentage of water retention. Scaffold samples were prepared into 2 × 2 cm squares, and the initial dry weights were recorded. Each sample was then immersed in deionized water for 6 h. After 6 h, excessive water was removed using blotting paper. The final weight of each scaffold was measured. Water retention capacity was calculated by percent weight change using the following equation:

Water Retention (%) = [(W_final_−W_initial_)/W_initial_] × 100 (2)

In this equation, W_final_ represents the weight of the scaffold after water immersion while W_initial_ represents the initial dry weight.

### 2.7. Scaffold Degradation

Scaffold samples were prepared into 2 × 2 cm pieces and the initial dry weights were recorded as a baseline. The samples were then immersed in PBS and incubated at 37 °C in a humidified atmosphere containing 5% CO_2_ for up to 28 days. At predetermined time points (7, 14, and 28 days), the samples were air-dried, and final dry weights were measured and calculated the percentage weight change using Equation (3). Morphological changes were observed using SEM, and quantitative analysis of fiber diameter and porosity was performed using the same protocol described in Section 2.3.

Weight change (%) = [(W_final_−W_initial_)/W_initial_] × 100 (3)

In this equation, W_final_ represents the weight of the scaffold after PBS immersion and air dry while W_initial_ represents the initial dry weight.

### 2.8. Cell Adhesion Assay

#### 2.8.1. Isolation and Culture of hAPCs

hAPCs were isolated from healthy, non-carious third molars extracted from patients aged 19–21 years (*n* = 3), with no clinical signs of infection. The study protocol was approved by the Human Experimental Committee of the Faculty of Dentistry, Chiang Mai University, Thailand (Approval No. 15/2024). Prior to extraction, patients rinsed with a 0.12% chlorhexidine mouthwash to reduce oral microbial load. Immediately after extraction, apical papilla tissues were carefully dissected using sterile instruments and enzymatically digested in a solution containing collagenase I and dispase II for 45 min at 37 °C. The resulting cell suspensions were cultured in complete alpha-minimum essential medium, supplemented with 10% fetal bovine serum, 1% penicillin–streptomycin, and 100 μmoL/L l-ascorbic acid. Cultures were maintained at 37 °C in a humidified atmosphere with 5% CO_2_. Cells of passage 2 were used.

#### 2.8.2. DAPI Staining and Cell Counting

PC-BG04 was chosen as a representative of the BG-coated scaffold compared to PC. Scaffolds were prepared into 2 × 2 cm pieces, sterilized using ethylene oxide gas and placed into individual wells of 24-well culture plates, pre-incubated with 500 μL of complete alpha-minimum essential medium at 37 °C with 5% CO_2_ for 24 h prior to the experiment. On the experiment day, excessive media was removed. hAPCs were seeded onto each scaffold at a density of 20,000 cells per well in a total volume of 1 mL complete medium, performed in triplicate. At 1 and 48 h post-seeding, the excess culture medium was removed, and the scaffolds were rinsed with PBS to eliminate non-adherent cells. Samples were then fixed with 4% paraformaldehyde for 15 min at room temperature, followed by permeabilization with 0.1% Triton X-100 for 10 min. Nuclei were stained with DAPI (1 μg/mL) for 15 min at room temperature in the dark. Excess dye was removed by PBS washing, and fluorescence imaging was performed using a fluorescence microscope (DMi8, Leica Microsystems, Buffalo Grove, IL, USA) across five randomly selected fields per sample. Quantitative analysis was conducted by counting DAPI-stained nuclei using ImageJ software (Version 1.54g, NIH, Bethesda, MD, USA).

## 3. Results

### 3.1. Scaffold Morphology

**Figure 1:**
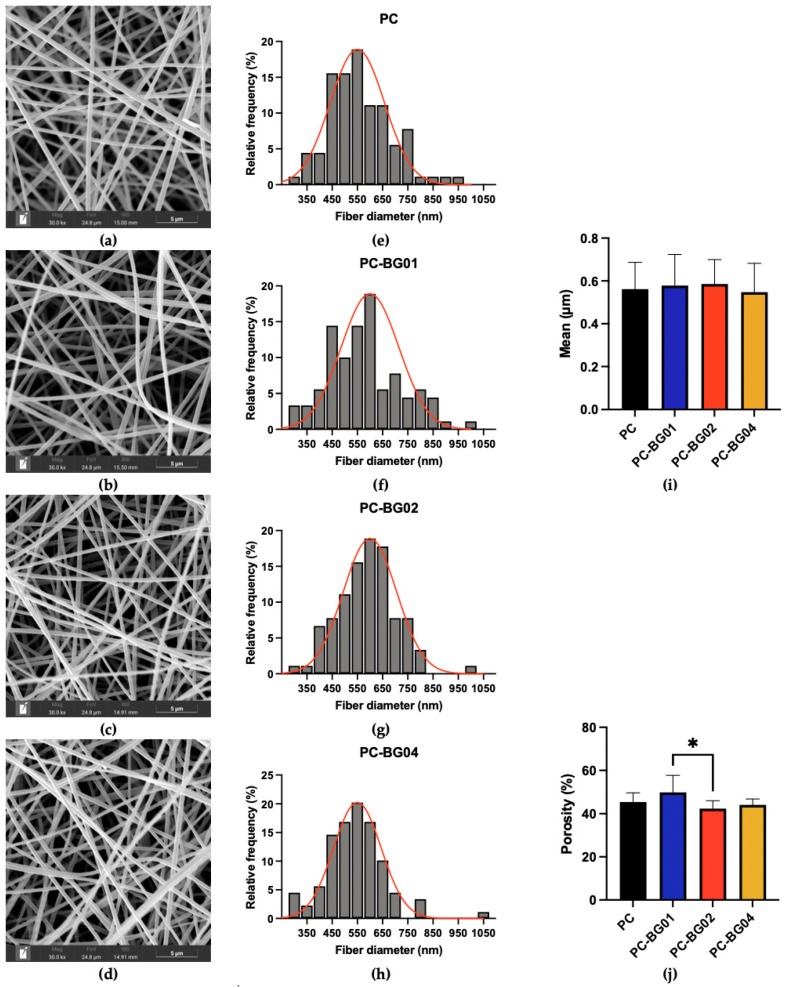
SEM images of (**a**) PC, (**b**) PC-BG01, (**c**) PC-BG02, and (**d**) PC-BG04 scaffolds (scale bar: 5 µm) corresponding with histogram of fiber diameter distributions (**e**–**h**), the red line represents the fitted distribution curve of fiber diameters. (**i**) Average fiber diameters of scaffolds (*n* = 90, mean ± SD). (**j**) Percentage porosity of scaffolds (*n* = 9, mean ± SD). * Indicates statistically significant differences between groups (*p* < 0.05).

The SEM images of the scaffolds are presented in Figure 1a–d. All groups exhibited a similar morphology characterized by smooth surface texture, cylindrical shape, and randomly oriented fiber alignment. Fiber diameter and porosity were analyzed from SEM images using ImageJ software. The fiber diameter distributions are presented in Figure 1e–h. The average fiber diameters of PC, PC-BG01, PC-BG02, and PC-BG04 were 561.6 ± 125.5 nm, 578.0 ± 145.7 nm, 585.9 ± 113.8 nm, and 547.3 ± 134.7 nm, respectively. No statistically significant differences in fiber diameter were observed among the groups (*n* = 90, *p* > 0.05, Kruskal–Wallis test) (Figure 1i). In contrast, porosity analysis revealed a significant difference between PC-BG01 (49.91 ± 7.76%) and PC-BG02 (42.37 ± 3.63%) (*n* = 9, *p* < 0.05, Kruskal–Wallis test). However, no significant differences in porosity were observed between PC (45.41 ± 4.28%) and any of the BG-coated groups (PC-BG01, PC-BG02, and PC-BG04: 44.13 ± 2.72%) (Figure 1j).

**Figure 2:**
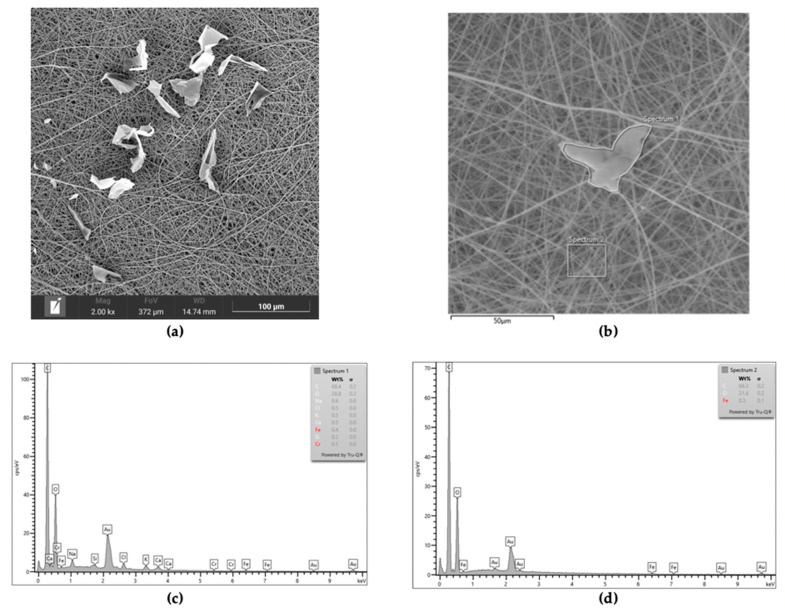
(**a**) SEM image of PC-BG04 scaffold showing surface-deposited beta-glucan particles (scale bar: 100 µm). (**b**) SEM image of the PC-BG04 scaffold indicating areas selected for elemental analysis using EDS (scale bar: 50 μm). (**c**) EDS spectrum of the particle area indicating the presence of sodium, consistent with the beta-glucan solution composition. (**d**) EDS spectrum of the surrounding area showing no sodium signal.

In all of the scaffolds incorporating BG (PC-BG01, PC-BG02, and PC-BG04), the flat particulate structures were observed on the surface of the fibers. The EDS spectra revealed the presence of sodium ions corresponding to the sodium hydroxide used as the solvent in the beta-glucan solution in the particulate areas, whereas sodium was not detected in other regions of the scaffold (Figure 2).

### 3.2. Fourier-Transform Infrared (FTIR) Spectroscopy

**Figure 3:**
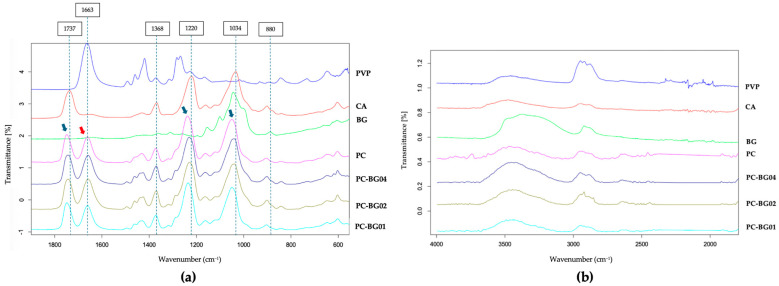
FTIR spectra of PVP/CA blended scaffold (PC), and BG-coated scaffolds (PC-BG01, PC-BG02, and PC-BG04), along with raw materials (PVP, CA, and BG powders). (**a**) Spectra from 2000 to 400 cm^−1^ and (**b**) spectra from 4000–1500 cm^−1^.

The FTIR spectra of PC, PC-BG01, PC-BG02, and PC-BG04, and the raw materials (PVP, CA, and BG) are presented in Figure 3. The spectrum of CA exhibited characteristic peaks at 1737 cm^−1^ (C=O stretching of the acetyl group), 1368 cm^−1^ (C–H bending of CH_3_), 1220 cm^−1^ (C–O stretching of the acetyl group), and 1034 cm^−1^ (C–O–C stretching of the polysaccharide backbone), while PVP displayed a prominent peak at 1663 cm^−1^, corresponding to the C=O stretching of the pyrrolidone ring. In the PC scaffold, the peaks associated with CA shifted to 1747, 1373, 1237, and 1049 cm^−1^, respectively (indicated by blue arrows), while the PVP peak was slightly shifted to 1661 cm^−1^ (indicated by a red arrow), suggesting possible chemical interactions between the two polymers.

The FTIR spectrum of BG showed distinct absorption peaks at 880 cm^−1^ (β-glycosidic linkage), and 1044 cm^−1^ (C–O–C stretching). In the BG-coated scaffolds, the β-glycosidic linkage signal was no longer observed, and C–O–C region in the backbone overlapped with that of CA (1043–1048 cm^−1^). The absence of the BG characteristics in the BG-coated scaffold may be attributed to the relatively low content of BG, limiting spectral detectability.

### 3.3. Beta-Glucan Quantitative Analysis

**Figure 4:**
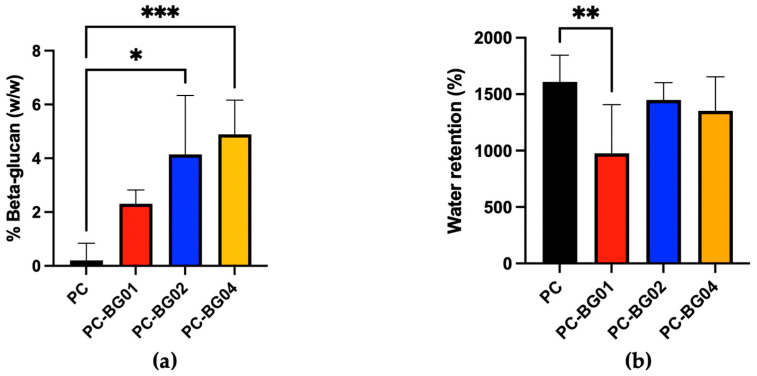
(**a**) Quantification of beta-glucan content in scaffolds, expressed as percentage weight per weight (% *w*/*w*) (mean ± SD, *n* = 3). (**b**) Water retention capacity of the scaffolds. The percentage of water absorbed relative to dry scaffold weight (mean ± SD, *n* = 9). Statistical significance between groups is indicated as * (*p* < 0.05), ** (*p* < 0.01), and *** (*p* < 0.001).

The beta-glucan detected values for PC, PC-BG01, PC-BG02, and PC-BG04 were 0.20 ± 0.64%, 2.31 ± 0.51%, 4.10 ± 2.20%, and 4.89 ± 1.27%, respectively (*n* = 3). Statistically, PC-BG02 and PC-BG04 contained significantly higher levels of beta-glucan compared to the PC group (*p* < 0.05, Kruskal–Wallis test), while no significant differences were observed among the BG-coated groups (Figure 4a).

### 3.4. Water Absorption Capacity

The measured water absorption percentages for PC, PC-BG01, PC-BG02, and PC-BG04 were 1610.0 ± 235.5%, 975.7 ± 432.4%, 1447.0 ± 153.5%, and 1352.0 ± 301.6%, respectively. A statistically significant difference was observed between the PC and PC-BG01 groups (*n* = 9, *p* < 0.05, Kruskal–Wallis test) (Figure 4b).

### 3.5. Scaffold Degradation

**Figure 5:**
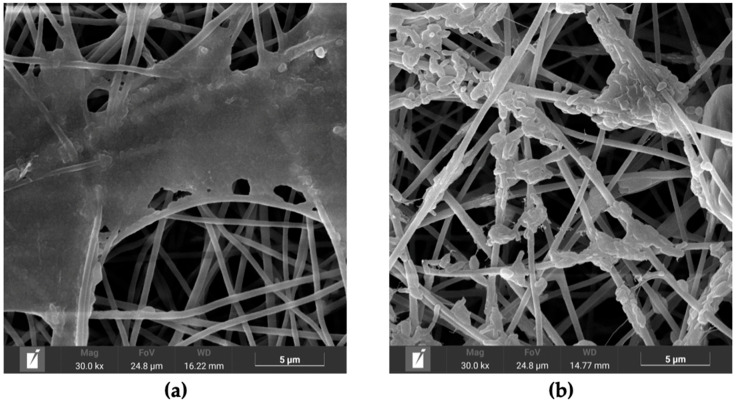
Degradation of beta-glucan particles over time. (**a**) SEM image of PC-BG01 scaffold after PBS immersion for 14 days (scale bar: 5 µm), showing partial degradation of beta-glucan particles with flattened morphology localized to the coated regions. (**b**) SEM image of PC-BG01 scaffold after 28 days (scale bar: 5 µm), showing advanced degradation of beta-glucan with irregular structures, while the surrounding scaffold matrix remains intact.

All groups exhibited similar morphological characteristics following immersion. The fibers became more compact due to structural collapse induced by the wet–dry process, resulting in reduced inter-fiber spacing. The beta-glucan particle on PC-BG01, PC-BG02, and PC-BG04 began to disintegrate at 14 and 28 days. At day 14, beta-glucan particles exhibited partial surface disintegration, appearing flattened or softened while remaining distinguishable from the surrounding scaffold structure (Figure 5a). By day 28, these regions showed more extensive degradation, with beta-glucan particles appearing collapsed, irregular, and more diffusely spread along the fiber surface (Figure 5b). These morphological changes were observed exclusively in areas where beta-glucan had been deposited, suggesting partial dissolution or degradation of the beta-glucan coating under the immersion conditions.

**Figure 6:**
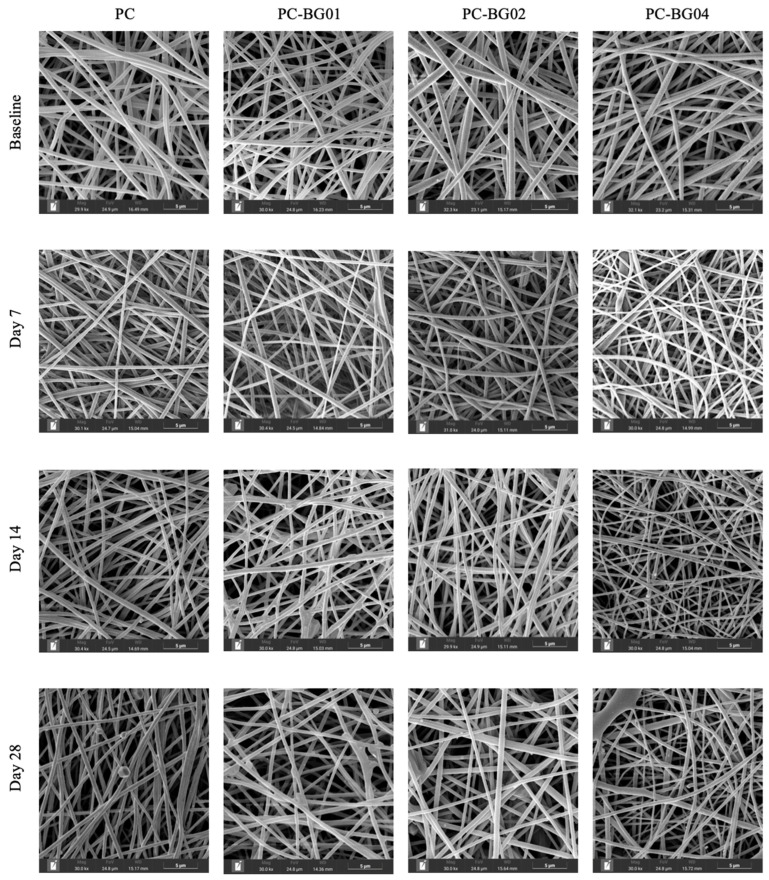
SEM images of scaffolds from all groups at different time points (baseline, 7, 14, and 28 days) after immersion in PBS and air-drying. Images were captured at comparable magnifications. Scale bar: 5 µm.

**Figure 7:**
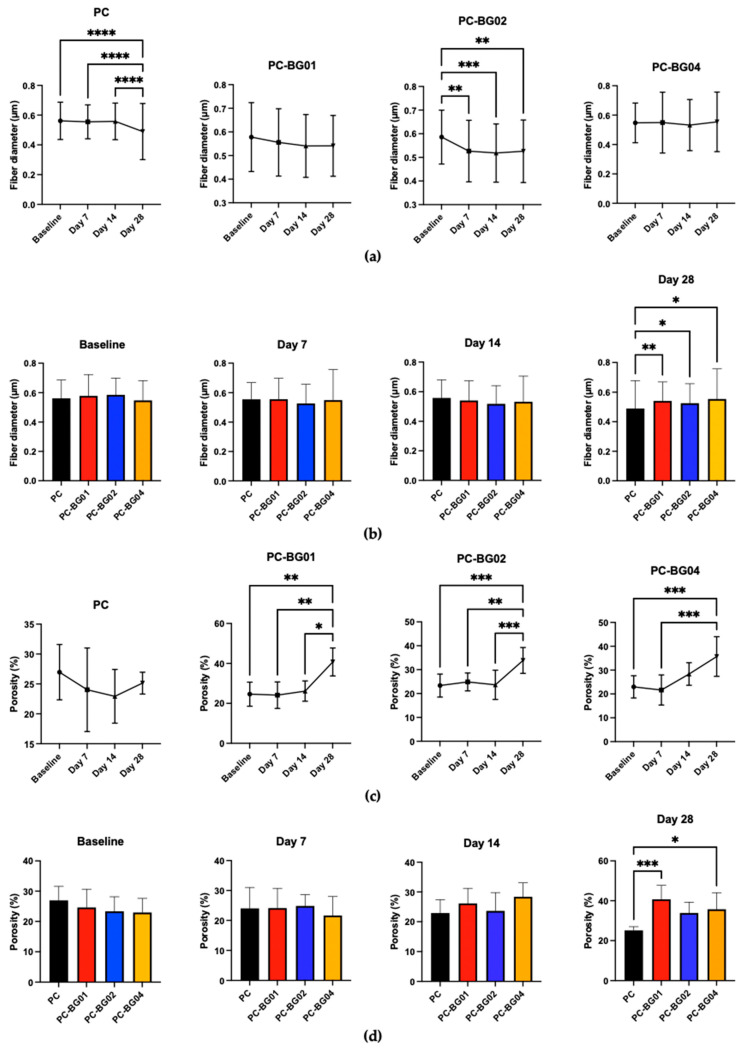

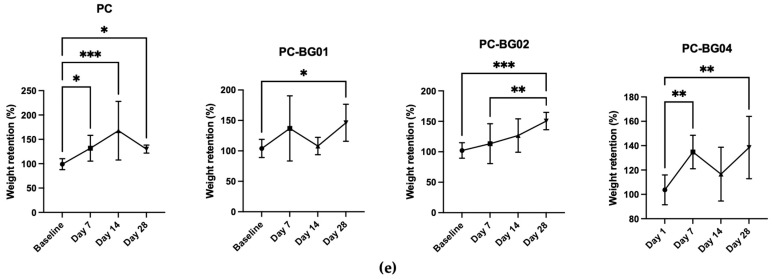
Physical changes in PC and BG-coated scaffolds over 28 days of PBS immersion followed by air-drying. (**a**) Fiber diameter distribution at each time point (baseline, 7, 14, and 28 days) (mean ± SD, *n* = 90). (**b**) Average fiber diameter across groups over time (mean ± SD, *n* = 90). (**c**) Porosity measurements at each time point (mean ± SD, *n* = 9). (**d**) Time-dependent changes in porosity for each scaffold group (mean ± SD, *n* = 9). (**e**) Dry weight changes in scaffolds after immersion (mean ± SD, *n* = 9). Statistical significance between groups is indicated as * (*p* < 0.05), ** (*p* < 0.01), *** (*p* < 0.001), and **** (*p* < 0.0001).

At the 7-day time point, all groups maintained smooth surface texture, cylindrical morphology, and random orientation. However, by days 14 and 28, the appearance of crystalline structures on the fiber surface was observed (Figure 6). Additionally, elemental analysis using EDS revealed the presence of sodium, carbon, oxygen, and phosphorus within the crystalline structures. Quantitative analysis demonstrated a gradual decrease in fiber diameter over time across all groups. Statistically significant reductions were observed at day 28 in the PC group and at day 7 in the PC-BG02 group (*n* = 90, *p* < 0.05, Kruskal–Wallis test) (Figure 7a). Intergroup comparisons at each time point revealed a significant difference in fiber diameter at day 28, where the PC group showed a smaller average diameter compared to the BG-coated groups (PC-BG01, PC-BG02, and PC-BG04) (Figure 7b).

Porosity analysis over time revealed that all BG-coated groups exhibited a significant increase in porosity at day 28 (*n* = 9, *p* < 0.05, Kruskal–Wallis test), whereas the PC group showed no significant changes at any time point (Figure 7c). Similarly, intergroup comparisons at each time point showed that at day 28, the PC group had significantly lower porosity than the BG-coated scaffolds, while no significant differences were observed at earlier time points (Figure 7d).

Dry weight of the scaffolds was measured at each time point following complete air-drying. Both the PC and PC-BG04 groups showed a significant increase in dry weight within the first 7 days (*n* = 9, *p* < 0.05, Kruskal–Wallis test), with no further significant changes from day 7 to day 28. The PC-BG01 group exhibited a significant increase in dry weight at day 28 compared to baseline, with no other significant differences. In the PC-BG02 group, dry weight significantly increased at day 28 compared to both baseline and day 7 (Figure 7e).

### 3.6. Cell Adhesion

**Figure 8:**
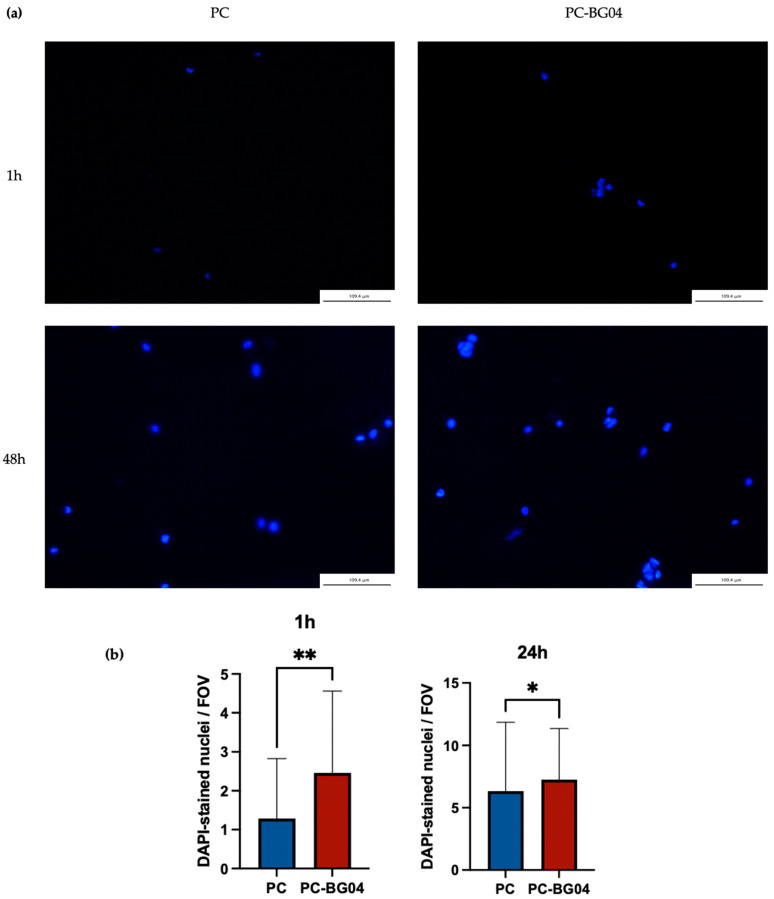
(**a**) DAPI-stained nuclei of hAPCs adhered to PC and PC-BG04 scaffolds at 1 and 48 h, demonstrating the scaffold’s ability to support cell attachment (scale bar: 109.4 µm). (**b**) Quantification of DAPI-stained nuclei adhered to PC and PC-BG04 scaffolds at 1 h and 24 h (mean ± SD, *n* = 3). Statistical significance between groups is indicated as * (*p* < 0.05), and ** (*p* < 0.01).

DAPI staining confirmed the ability of hAPCs attaching to both PC and PC-BG04 scaffolds surfaces at both 1 and 48 h post-seeding. At 1 h, scattered nuclei were observed, indicating initial cell attachment. By 48 h, a greater number of nuclei were visible on the scaffold surface, suggesting continued cell retention or proliferation over time (Figure 8a). Quantitative analysis demonstrated the significant increase in cell attachment on the BG-coated scaffold compared to the PC scaffold in both 1 and 24 h (*n* = 3, *p* < 0.05, Kruskal–Wallis test) (Figure 8b).

## 4. Discussion

The present study introduces a novel approach to fabricating a scaffold incorporating beta-glucan in an easy-to-use format suitable for intracanal application in REPs and provides preliminary data for scaffold characterization. The scaffolds present smooth fibers with high porosity accompanied by high hydrophilicity. Although the presence of BG was undetectable by FTIR spectra, its successful incorporation was confirmed by elemental and enzymatic quantitative analyses. Biologically, the scaffold provided a favorable surface for the initial attachment of hAPCs, particularly at the BG loading of 0.4 mL. These findings suggest the potential of BG-coated PVP/CA scaffolds as a bioactive platform to support dental tissue regeneration.

Physiological characterization confirmed that all scaffolds maintained smooth fiber architecture without disruption following beta-glucan coating. Consistent with previous studies, the use of a 10% polymer concentration, an applied voltage of 15 kV, and a working distance of 10–15 cm resulted in uniform, cylindrical, bead-free scaffold [22,26]. When observing the fiber diameter changes over time, all scaffolds showed a reduction in fiber diameter after 28 days of immersion in Phosphate buffer saline (PBS), reflecting progressive structural degradation. Notably, the BG-coated scaffolds retained larger fiber diameters for a longer duration compared to the control, suggesting that beta-glucan may confer some protection against early scaffold erosion. This delayed reduction in fiber diameter may contribute to maintaining scaffold integrity during the early phases of tissue regeneration. Porosity is another critical factor influencing the suitability of scaffolds for tissue regeneration [33,34,35,36]. All of our scaffold formulations exhibited high porosity and substantial water absorbance capacity, which could support ECM-mimicking properties and promote cell interactions. Porosity increased markedly over 28 days in the BG-coated groups compared to the PC group. This finding may reflect degradation of BG particles on the scaffold surface, even though fiber diameter remained more stable in the BG-coated scaffolds. The dry weight changes also indicated an increase in scaffold mass at all time points after immersing with PBS, accompanied by the formation of crystalline structures on the scaffold surface, particularly at 28 days. Elemental analysis suggests the possible formation of phosphate salts on the scaffold, which may account for the observed weight gain. In contrast, hydroxyapatite crystallization on PVP/CA scaffolds has been reported when immersed in simulated body fluid containing calcium ions [25], highlighting the potential of PVP/CA-based scaffolds for promoting hard tissue formation.

Chemical characterization of the scaffolds confirmed beta-glucan existence in the scaffold by EDS mode in the SEM image and BG enzymatic assay. EDS analysis confirmed the presence of sodium ions on beta-glucan-suspected particles, while no sodium signal was detected in other areas of the scaffolds. The detected sodium originates from sodium hydroxide, which is used as the solvent for the BG solution, and it serves as a distinguishing element from the polymer matrix. Beta-glucan content was further quantified using an enzymatic assay. A very small amount of beta-glucan was detected in the PC scaffold as a consequence of partial hydrolysis of cellulose in the CA backbone, whereas the BG-coated scaffolds (PC-BG02 and PC-BG04) exhibited significantly higher beta-glucan content. In contrast, FTIR analysis did not clearly reveal distinct characteristic absorption peaks for BG in the BG-coated scaffolds. The BG powder typically exhibits an absorption peak at 880 cm^−1^, corresponding to the β-glycosidic bond [37], but this peak is absent in BG-coated scaffolds. The 1044 cm^−1^ peak corresponding to C–O–C stretching on the beta-glucan backbone was also detected but superimposed with the C–O–C stretching band of the polysaccharide backbone of CA, complicating interpretation. Furthermore, the BG content quantified enzymatically was low (2.31–4.89%), potentially contributing to weak or ambiguous FTIR signals. Overall, these results suggest that although BG was incorporated, its low concentration and overlapping peaks with core polymers limit the ability of FTIR to clearly confirm its presence and interaction in the scaffold [38]. Notably, characteristic absorption bands of CA (from 1737, 1368, 1220, and 1034 cm^−1^) showed blue shifted to 1747, 1373, 1237, and 1049 cm^−1^ in the PC scaffold, as well as a shift in the PVP from 1651 to 1661 cm^−1^, suggest possible chemical interactions between the two polymers causing from changing in the electronic environment that enhance the bond’s vibrational energy [39]. However, the nature of these interactions between PVP and CA during the electrospinning process remains inconclusive. The result of this study is consistent with previous findings describing interactions between functional groups of PVP and CA [22,26]. In contrast, other studies have reported the coexistence of PVP and CA without clear evidence of molecular interaction [40,41].

The biological aspect of the scaffolds was evaluated by the adhesion ability of hAPCs. Based on preliminary biocompatibility screening (Figure S1), scaffolds incorporating more than 0.5 mL of BG solution exhibited cytotoxicity. Therefore, three formulations with lower volumes (0.1, 0.2, and 0.4 mL) were developed, and PC-BG04 was selected for biological comparison with the PC scaffold. Fluorescence staining confirmed the presence of hAPCs attached to both scaffold surfaces at 1 and 48 h post-seeding. Quantitative analysis resulted in a significant increase in cell attachment in the PC-BG04 group at both time points, suggesting improvement of initial cell attachment and retention by BG. This observation is consistent with previous studies reporting the bioactive role of beta-glucan in promoting cellular responses in hAPCs and other cell types [14,20,42,43,44,45,46]. Further investigation is required to determine whether these effects translate into enhanced downstream outcomes such as proliferation, migration, odontogenic differentiation, or mineralized tissue formation.

Nonetheless, the experimental conditions did not replicate the inflammatory microenvironment commonly present in immature permanent teeth undergoing REPs. In addition, other important aspects of biological evaluation could not be addressed in this study, such as cell proliferation, migration, and, most importantly in the regenerative context, odontogenic differentiation and mineralization. Future studies should specifically investigate scaffold performance in promoting odontogenic differentiation and mineralized tissue formation, both in vitro and in vivo. However, these results introduce the PVP/CA with coated BG scaffold as a potential scaffold in REPs. Further studies may investigate and re-optimize this type of scaffold for other applications, initially focusing on specific cell types or treatments such as wound dressing, bone regeneration, and dental therapies like vital pulp treatment. In addition, incorporating other drugs or bioactive molecules in combination with PVP/CA may provide additional benefits for future applications in REPs.

## 5. Conclusions

This study successfully coated BG particles on the PVP/CA electrospun scaffold with electrospraying technique. This novel scaffold demonstrated favorable physical properties with effective BG surface deposition on blended PC scaffold. BG particles degradation was observed at 14 and 28 days with scaffold integrity and mineral deposition. Biologically, the BG-coated scaffold significantly enhanced initial hAPCs adhesion compared to the control. These findings highlight the potential of BG-coated PVP/CA scaffolds as bioactive scaffolds for REPs.

## Figures and Tables

**Table 1:** Composition of scaffolds selected for further scaffold characterization.

Scaffold Formula	PVP (% *w*/*v*)	CA (% *w*/*v*)	Volume of BG Solution Sprayed (mL)
PC	3.0	7.0	-
PC-BG01	3.0	7.0	0.1
PC-BG02	3.0	7.0	0.2
PC-BG04	3.0	7.0	0.4

All scaffolds were fabricated from 10% (*w*/*v*) PVP/CA polymer solution. Beta-glucan (BG) was dissolved at 8% (*w*/*v*) in 1 M NaOH and electrosprayed onto PC scaffold.

## Data Availability

The data presented in this study are available upon reasonable request from the corresponding author.

## Supplementary Materials

The following supporting information can be downloaded at: https://www.mdpi.com/article/10.3390/polym17192693/s1, Figure S1: Percentage difference (mean ± SD) in proliferation of human apical papilla cells (hAPCs) at 24, 48, and 72 h after seeding on electrospun scaffolds containing beta-glucan (BG) at 0.25, 0.5, and 1 mL, compared with PC scaffolds.

## Abbreviations

The following abbreviations are used in this manuscript:

REPs	Regenerative endodontic procedures
hAPCs	Human apical papilla cells
BG	Beta-glucan
ECM	Extracellular matrix
PVP	Polyvinylpyrrolidone
CA	Cellulose acetate
DMAc	N,N-dimethyl acetamide
SEM	Scanning electron microscope
EDS	Energy-dispersive X-ray spectroscopy
FTIR	Fourier transform infrared
PBS	Phosphate buffer saline
DAPI	4′,6-diamidino-2-phenylindole dihydrochloride
