# Supplementary material for: Electrospray Beta-Glucan Particle Coated PVP/CA Electrospun Mat as a Potential Scaffold for Dental Tissue Regeneration
Source: Polymers (Basel). 2025 Oct 5;17(19):2693. doi: 10.3390/polym17192693 (PMC12526963; doi:10.3390/polym17192693)
Supplement: Supplementary file 1 [file polymers-17-02693-s001.zip › polymers-3872838-supplementary.pdf]

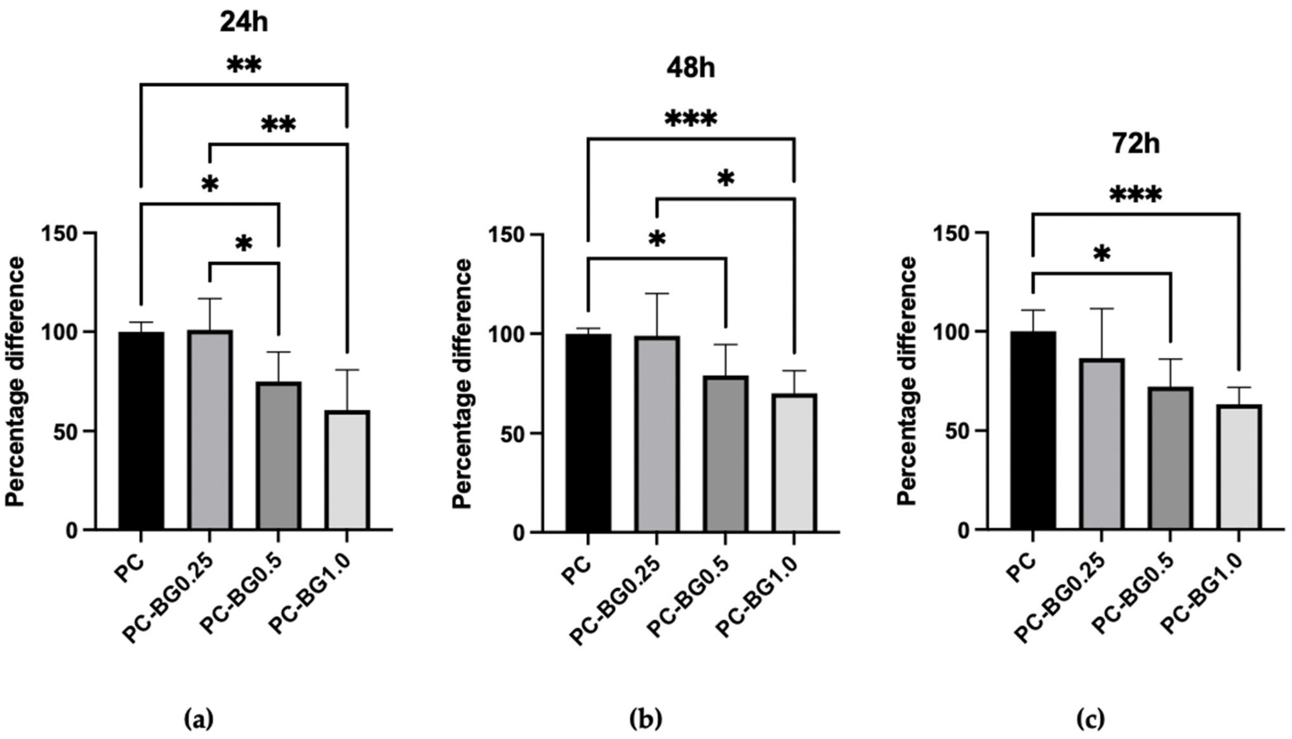

Figure S1. Percentage difference in proliferation of human apical papilla cells (hAPCs) at 24 (a), 48 (b), and 72 (c) hours after seeding on electrospun scaffolds containing beta-glucan (BG) at 0.25, 0.5, and 1 mL, compared with PC scaffolds (mean ± SD, n = 3). Statistical significance between groups is indicated as * ($p < 0.05$), ** ($p < 0.01$), and *** ($p < 0.001$).
